# Supplementary material for: Chromothripsis during telomere crisis is independent of NHEJ, and consistent with a replicative origin
Source: Genome Res. 2019 May;29(5):737–49. doi: 10.1101/gr.240705.118 (PMC6499312; doi:10.1101/gr.240705.118)
Supplement: Supplemental Material [file supp_gr.240705.118_Supplemental_file_1.zip › contigs/annotated_contigs/DB51/contig.2.DB51_length_312_mean_cov_4.80769230769.docx]

**DB51_length_312_mean_cov_4.80769230769**

```
ATTGATTATCCTCAGGAGCCACCCCCAACGCCCCTGTTTGCTTCTAGATCTATAACTAGACTAAAGTCATGGCGGGTCCCTGGAGGTAA
 >chr4:133277198-133277449 - E=4e-137 p=5e-03
GGTTGAGAGTGTGACCCATGAGGAGGTACATTACACTTGAAAAGAACACTTGAAAAGAACTGCTCGAGTTATCTAATTTATATAAACAG

AAATCTGGAGAACAGGCATGGAAATGGATATTAAGGGTATAGGATAATGGTGGAAGGCACAGAG|TTTGATCAG|CAGCTTGGTTACTG
                                                                 >chr12:95288007-952880
GAATTTAATAAACTAATTGGAGAATGGTGTAAGTGCAAGATAACAGC77 + E=1e-27
```
